# Natural Disease Resistance in Threatened Staghorn Corals

**DOI:** 10.1371/journal.pone.0003718

**Published:** 2008-11-13

**Authors:** Steven V. Vollmer, David I. Kline

**Affiliations:** 1 Marine Science Center, Northeastern University, Nahant, Massachusetts, United States of America; 2 Smithsonian Tropical Research Institute, Balboa, Panama; 3 Centre for Marine Studies, University of Queensland, Queensland, Australia; University of North Carolina at Chapel Hill, United States of America

## Abstract

Disease epidemics have caused extensive damage to tropical coral reefs and to the reef-building corals themselves, yet nothing is known about the abilities of the coral host to resist disease infection. Understanding the potential for natural disease resistance in corals is critically important, especially in the Caribbean where the two ecologically dominant shallow-water corals, *Acropora cervicornis* and *A. palmata,* have suffered an unprecedented mass die-off due to White Band Disease (WBD), and are now listed as threatened under the US Threatened Species Act and as critically endangered under the IUCN Red List criteria. Here we examine the potential for natural resistance to WBD in the staghorn coral *Acropora cervicornis* by combining microsatellite genotype information with *in situ* transmission assays and field monitoring of WBD on tagged genotypes. We show that six percent of staghorn coral genotypes (3 out of 49) are resistant to WBD. This natural resistance to WBD in staghorn corals represents the first evidence of host disease resistance in scleractinian corals and demonstrates that staghorn corals have an innate ability to resist WBD infection. These resistant staghorn coral genotypes may explain why pockets of *Acropora* have been able to survive the WBD epidemic. Understanding disease resistance in these corals may be the critical link to restoring populations of these once dominant corals throughout their range.

## Introduction

Disease epidemics have radically altered tropical coral reefs and are becoming more frequent and extensive because of climate change [1]–[3]. This is most apparent in the Caribbean where diseases have caused massive and widespread die-offs of the key herbivorous sea urchin *Diadema anitillarum* [4], common Gorgonian sea fans [5], [6] and the two ecologically dominant shallow-water corals–the staghorn coral *Acropora cervicornis* and the elkhorn coral *A. palmata* [7], [8]. The Caribbean-wide mass die-offs of both the shallow-water *Acropora* corals and the keystone urchin *D. antillarium*, in particular, have been major contributors to the rapid decline of Caribbean coral reefs and the dramatic phase shift from coral to macroalgal dominance [7], [9], [10].

Reef-building corals, in general, have been susceptible to the global rise in marine diseases [1], [11], [12]. As foundation species on tropical reefs, the impacts of disease on corals can ripple throughout the ecosystem [7], [11]. The effect of the White Band Disease (WBD) epidemic on the Caribbean *Acropora* corals demonstrates the ecosystem-level impacts of coral disease on tropical reefs [7]. Since it was first observed in the late 1970s [8], WBD has caused unprecedented Caribbean-wide declines in its hosts *A. cervicornis* and *A. palmata* [7], [13], [14], with losses of up to 95% of living acroporid cover common across the greater Caribbean [13], [15], [16]. Recovery of these formerly dominant shallow-water corals has been slow [7], [17], due in large part to poor larval recruitment [18]–[20], highly restricted larval dispersal [21], [22] and a heavy reliance on asexual (i.e. vegetative) propagation [23]–[25]. As a result, both species have recently been listed as threatened on the US Endangered Species Act [26], [27] and listed as critically endangered under the International Union for the Conservation of Nature (IUCN) Red List criteria [28].

**Figure 1:**
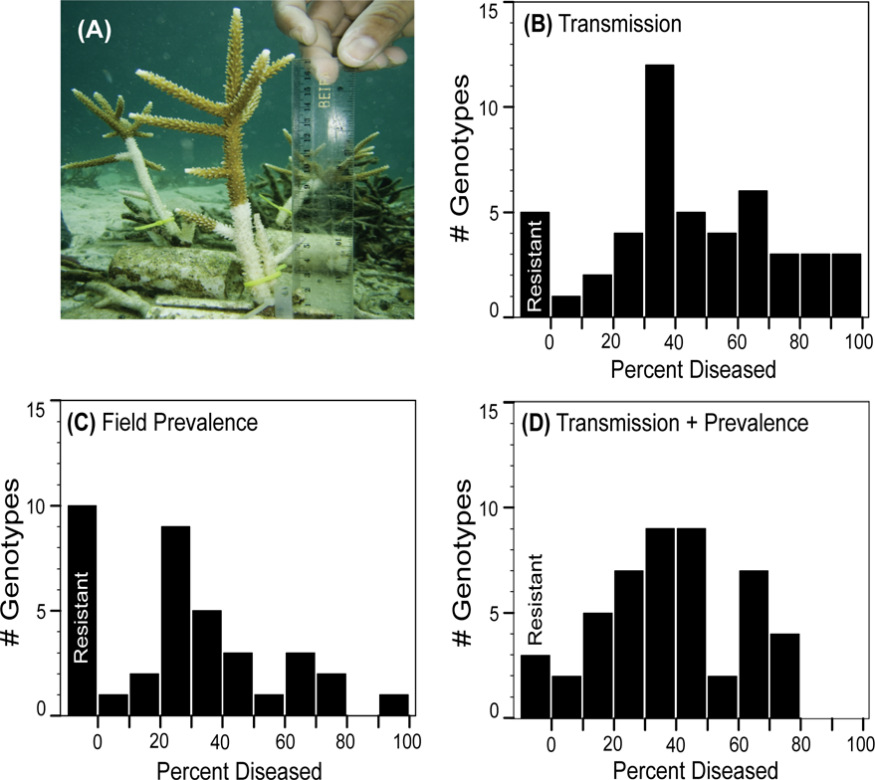
Resistance to White Band Disease (WBD) in the staghorn coral *Acropora cervicornis*. (A) WBD transmission to a coral fragment occurs rapidly as illustrated by the progress of the advancing white band of disease after three days of direct contact (grafting) with an infected coral fragment. (B) *In situ* transmission experiments identified five staghorn coral genotypes that did not contract WBD. (C) Field surveys of WBD prevalence identified ten genotypes that were not observed in the field with WBD. (D) Integrated field surveys and experimental transmission results show that three staghorn coral genotypes were resistant to WBD infection.

Yet, despite its dramatic impacts, much about the etiology and ecology of WBD remains poorly understood [7], [11]. WBD draws its name from its appearance as a rapidly advancing white band of diseased tissue [8], [29] (Fig. 1A). WBD appears to be host-specific, infecting only the Caribbean *Acropora* [11]. It has two forms–WBD type I which is ubiquitous throughout the Caribbean and WBD type II which has been described from the Bahamas [29]–and can be transmitted via direct contact and through vectors such as the corallivorous snail *Corallophyllia abbreviata* [30]. The WBD pathogen has not been isolated in pure culture, but histological and genetic data suggest that the pathogen is bacterial [29], [31]–[33]. Recent genetic surveys indicate that a marine *Rickettsia* bacterium is associated with WBD type I [31] while the bacterium *Vibrio charcharia* appears to be associated with WBD type II [29]. Nothing is known about the potential for host resistance to WBD in the Caribbean *Acropora* species.

Here we assess the potential for natural resistance to WBD in the threatened staghorn coral *Acropora cervicornis*. To do this, WBD resistance was assayed on 49 staghorn coral genotypes from four populations in Bocas del Toro, Panama using a series of *in situ* transmission experiments and field monitoring of WBD prevalence. We show that 3 out of the 49 staghorn coral genotypes assayed were naturally resistant to WBD.

## Results

A total of 106 staghorn coral colonies were tagged in four populations (i.e. reefs) from Bocas del Toro, Panama and then genotyped using five microsatellite loci in order to identify unique staghorn genotypes from clones produced by asexual fragmentation. Data from the five microsatellite loci identified 49 out of the 106 samples as unique staghorn coral genotypes (Table 1) or 46% of the sample. Each population possessed a relatively high number of genotypes; the number of genotypes (genets) per population ranged from 7 at Punta Caracol to 17 at Salt Creek. The Punta Caracol and Casa Blanca populations had fewer genets per sample (G/N) and lower clonal diversity indices (Ds and Evenness) than the Crawl Cay and Salt Creek populations (Table 1).

**Table 1:** Number of staghorn coral samples (N), unique genotypes or genets (G), and the ratio of genets per sample (G/N) per population along with clonal diversity (D_s_), its evenness (E), and the number of genets with clones (N_cg_).

Population	N	G	G/N	D_s_	E	N_cg_
Punta Caracol	24	7	0.292	0.558	0.307	3
Casa Blanca	35	12	0.343	0.699	0.260	4
Crawl Cay	23	13	0.565	0.850	0.411	3
Salt Creek	24	17	0.708	0.938	0.584	3
Total	106	49	0.462			

In order to assay WBD resistance on our staghorn coral genotypes, four sets of *in situ* transmission experiments were conducted (in July 2005, Sept. 2005, May 2006 and Aug. 2006) in parallel across the four sites. WBD transmission was achieved by grafting active fragments of WBD to replicate fragments of each staghorn coral genotype placed on clips in cinderblock common gardens (Fig. 1A). Transmission data was obtained for all 49 staghorn coral genotypes. For each genotype, WBD transmission was attempted five or more times (Fig. 1B). Overall, the rate of WBD transmission averaged 45.5% on the experimental fragments grafted with active WBD, whereas only 3.3% of the control fragments developed WBD. WBD on the control fragments was likely due to prior infection on the control fragments.

Data from the *in situ* transmission experiments show that the percentage of WBD infections per genotype varied substantially across the 49 assayed staghorn coral genotypes (Fig. 1B). Most of the staghorn coral genotypes (n = 12) fell within 30–40% disease class, while nine genotypes were highly susceptible to WBD with greater than 70% disease. Most importantly, five genotypes did not contract WBD (genotypes-sc78, sc81, sc89, sc96 and ck311), despite repeated attempts to transmit WBD to them [sc78 (n = 5 replicates), sc81 (6), sc89 (6), sc96 (6) and ck311 (9)]. These five WBD resistant genotypes were from two of the four populations; four genotypes were from Salt Creek (sc78, sc81, sc89, and sc96) and one was from Crawl Cay (ck311)]. We had sufficient replication (6 or more attempts) for four of these resistant genotypes (sc81, sc89, sc96, and ck311) to show a statistically significant degree of resistance [Pr<0.026 given the binomial probability Pr (No Infection) = (1–0.455)^6+^].

The prevalence of WBD on our tagged staghorn coral genotypes was surveyed in the field on four separate occasions (Sept. 2005, Dec. 2005, May 2006 and August 2006). WBD prevalence on our tagged genotypes averaged 28.2% across the four sites. The percentage of times WBD was observed on each tagged genotype in our field surveys was lower than in the *in situ* transmission experiments (Fig. 1) due to the lower prevalence of WBD in the field (28.2% versus 45.5%, respectively). A total of ten staghorn coral genotypes were never observed with WBD symptoms during our four field surveys (Fig. 1C).

We combined the data from the *in situ* transmission experiments (Fig. 1B) and field surveys (Fig. 1C) to calculate a conservative index of disease susceptibility (Fig. 1D). Three of the ten genotypes identified as resistant in the field also failed to contract WBD in our transmission experiments (sc78, sc96, ck311), and thus were fully resistant to WBD over the course of our study. The other two genotypes that we identified as resistant based on our transmission experiments (sc81 and sc89) were each observed with WBD once in the field. For the three genotypes that displayed resistance in both our field surveys and transmission experiments (sc78, sc96, ck311), the combined binomial probabilities for observing full WBD resistance (i.e. no infections) in both the transmission experiments and field surveys were significant (P = 0.043, P = 0.007, P = 0.001, respectively).

## Discussion

Data from our *in situ* transmission experiments and field surveys indicate that roughly six percent of staghorn genotypes (3 out of 49) from Bocas del Toro, Panama are resistant to WBD infection. This natural resistance to WBD in threatened staghorn corals provides the first evidence for host disease resistance in reef-building corals, and may explain why pockets of staghorn corals have survived the Caribbean-wide epidemic of WBD over the past thirty years.

Natural resistance to WBD in staghorn corals has important evolutionary and ecological implications for how staghorn coral populations may be responding to the WBD epidemic. In an evolutionary scenario akin to G. C. Williams (1975) strawberry-coral model of genotype selection [34], we predict that WBD resistant genotypes of staghorn coral will have a selective advantage over non-resistant genotypes, and thus should accumulate locally within populations over time via asexual, vegetative fragmentation. Staghorn corals are prolific vegetative fragmenters [19], [24], [35], and thus asexual propagation of WBD resistant staghorn genotypes within populations (i.e. reefs) should provide an effective means for the local recovery and persistence of staghorn coral populations where WBD resistant genotypes occur. It is possible that differences in the numbers of naturally resistant genotypes between reefs may explain for why some staghorn coral populations have fared better than others over the course of the WBD epidemic.

Ultimately, however, the broad-scale recovery of staghorn coral populations across the greater Caribbean will have to be achieved by the successful dispersal and recruitment of staghorn coral larvae; preferably those carry genetic variation for WBD resistance. This may prove to be the limiting step for staghorn coral recovery. Staghorn corals have historically been poor sexual recruiters [10], [20], [35]–[37], relying predominantly on localized asexual fragmentation instead [19], [23]–[25], and staghorn coral recruits continue to be rare on most Caribbean reefs [18], [38]. In addition, we know of no instances where a sexual recruitment pulse has resulted in the recovery of staghorn coral populations since the WBD epidemic. Instead, Caribbean *Acropora* populations appear to be experiencing recruitment failure [39], possibly augmented by Allee effects resulting from WBD-induced population reductions [25], [40], [41].

Even if successful larval recruitment events were to occur, the scale of over which staghorn larvae could reseed downstream reefs will be limited by their restricted dispersal potential. Genetic data indicate that larval dispersal in *A. cervicornis* [22] and its congener *A. palmata* [21] is geographically restricted across the Caribbean over spatial scales less than 500 kilometers [21], [22], [42], [43]. For *A. cervicornis*, the genetic data indicate that gene flow can be limited over spatial scales as small as adjacent reefs (i.e. 2–5 kilometers)[22]. The combination of poor sexual recruitment and geographically restricted gene flow in staghorn corals suggests that larval recruitment from healthy staghorn coral populations will not be sufficient to recover downstream reefs in the next few decades. Thus, the conservation and restoration of staghorn coral populations will have to be achieved through the local protection of remnant populations and aggressive strategies aimed at propagating and transplanting WBD resistant genotypes to reefs.

Our data show that staghorn corals display a wide range of phenotypic variation in their response to WBD, ranging from highly resistant to highly susceptible coral strains. A number of environmental and genetic factors are likely contributing to this phenotypic variation. Yet, the occurrence of WBD resistant genotypes suggests that this disease resistance has an underlying genetic basis. Given the close evolutionary relationship between *A. cervicornis* and *A. palmata* [its congener and hybrid partner [44]], it is likely that WBD resistance exists in *A. palmata* as well. Natural genetic variation for host disease resistance has been documented in a variety of animals and plants [45]–[48], including marine shrimp [49]–[52] and oysters [53]–[57]. While nothing is known about which genes might confer disease resistance in reef corals, genetic surveys for resistance genes (or R-genes) in other taxa [46], [47] indicate that the genetic basis of disease resistance often occurs on genes involved in pathogen recognition and innate immunity [46], [47]. Reef corals do possess key components of the invertebrate innate immune pathway, including Toll/Toll-like receptors [58], which might form the genetic basis for disease resistance in corals. Thus, future research on the genetic basis of disease resistance in corals has the potential to uncover the gene(s) involved in host-pathogen resistance and recognition and allow for surveys of resistant gene variants in disease impacted corals like the Caribbean *Acropora*.

Evidence for natural disease resistance in a reef-building corals supports growing interest in the role that host resistance might play in buffering the impacts of the global rise in marine diseases on tropical coral reefs and elsewhere [1], [6], [59], [60]. In addition to staghorn corals, disease resistance has been identified in a number of other marine taxa, including oysters [53], shrimp [49], [52] and abalone [61]. Rapid evolution of host genetic resistance has been evoked to explain sharp reductions in disease infections by the terrestrial fungus *Aspergillus* on Gorgonian soft corals in the Caribbean [6]. While it remains to be seen how important and pervasive disease resistance in reef corals might be, its existence demonstrates that some corals have the innate ability and adaptive genetic variation to respond to diseases and possibly other stressors, including coral bleaching. With coral bleaching, strong emphasis has been placed on the role of algal symbiont diversity as a means to respond to bleaching, i.e. the adaptive bleaching hypothesis [62]–[66]. Like host resistance to disease, we suggest that there is potential for adaptive genetic variation for bleaching resistance within the genomes of corals as well.

Clearly, more research is needed to elucidate the genetic and environmental factors underlying natural disease resistance in reef-building corals. The approach taken here to identify naturally resistant strains of staghorn corals demonstrates that disease resistant corals can be identified using relatively simple means and provide a stepping-off point for further research on the genetic basis of disease resistance, pathogen recognition and innate immunity in reef-building corals. From a conservation standpoint, these simple transmission assays provide a means for identifying disease resistant coral strains, which in combination with coral farming and local replanting of diverse sets of disease resistant coral genotypes, would provide an effective means to restore threatened *Acropora* populations throughout the Caribbean.

## Materials and Methods

### Identification of staghorn coral genotypes

Permanent transects with tagged, genotyped staghorn corals were established in four populations (Punta Caracol, Casa Blanca, Crawl Cay, and Salt Creek) in Bocas del Toro, Panama, in order to provide distinct staghorn coral genotypes for *in situ* WBD transmission experiments and to monitor WBD prevalence in the field. A total of 106 staghorn coral colonies were tagged with numbered aluminum tags along permanent transects in a 3×6 meter grid within each of the four populations. A small fragment (ca. 1 cm) of each coral was sampled, placed directly in Chaos DNA buffer (a concentrated guanidine thiocynate buffer), and the DNA later extracted using published protocols [67]. Each tagged coral was genotyped at five microsatellite loci [loci 166, 181, 182, 187, and 201; after [68]] using modified PCR protocols, and by multiplexing all five PCR products for genotyping on a ABI 3100xl capillary sequencers (Applied Biosystems) using 4-color fluorescent primer labels and Liz-500 size standard. The microsatellite data were scored using Genescan and Genotyper software (Applied Biosystems). The programs GenAElx ver. 6 [69] and GenoDive [70] were used to identify unique staghorn genotypes (and their clones) and calculate clonal diversity indices. Multilocus genotypes from these five microsatellite loci identified unique staghorn coral genotypes and their clonemates with high probabilities of identity (p<10^−5^), and no shared identical genotypes were observed between populations (i.e. reefs). Additional results from these microsatellite data will be published elsewhere as part of spatial genetic structure study on *A. cervicornis* (Vollmer, In Prep.).

### In situ WBD transmission

Four sets of *in situ* transmission experiments were conducted (in July 2005, Sept. 2005, May 2006 and Aug. 2006) in order to assay WBD resistance on our tagged staghorn coral genotypes. For each transmission experiment, four healthy coral fragments (ca. 30 cm long) were sampled from each staghorn coral genotype and placed into cinderblock common gardens on PVC clips located adjacent to each site. WBD transmission was attempted on three of the four fragments by grafting (i.e. cable-tying) active pieces of WBD sampled from diseased corals in the field; the fourth fragment was grafted with a healthy (i.e. asymptomatic) piece of staghorn coral as a control. The presence or absence of WBD on each fragment was then scored 3–5 days after transmission (Fig. 1A). WBD was readily apparent and identified as a mobile front of white disease tissue interface, and was easily differentiated from localized mortality due to non-self aggression reactions resulting from tissue grafts [19].

Data presented here are from staghorn coral genotypes with more than 5 replicate transmission attempts. WBD resistant staghorn coral genotypes were defined conservatively as genotypes where no WBD transmission was observed. The probability that this WBD resistance was significant was calculated using the binomial probability [i.e. Pr(No WBD Infection) = (1–Average rate of Transmission)^#Replicate Trials^]. WBD susceptible genotypes were defined arbitrarily as corals with greater than 70% WBD infection.

### Field Surveys

The prevalence of WBD was surveyed on the tagged coral genotypes in the field at each of the four study sites on four separate occasions (Sept. 2005, Dec. 2005, May 2006 and August 2006). These field data on WBD prevalence were used in combination with the transmission studies to verify WBD resistance on the assayed staghorn coral genotypes.
